# Advancing Pain Management Protocols for Intrauterine Device Insertion: Integrating Evidence-Based Strategies Into Clinical Practice

**DOI:** 10.7759/cureus.63125

**Published:** 2024-06-25

**Authors:** Eden Estevez, Shivaughn Hem-Lee-Forsyth, N'Diera Viechweg, Sharon John, Stephanie P Menor

**Affiliations:** 1 Obstetrics and Gynecology, St. George’s University School of Medicine, St. George, GRD; 2 Public Health, St. George's University, St. George, GRD; 3 Internal Medicine, St. George’s University School of Medicine, St. George, GRD; 4 Internal Medicine, St. George's University School of Medicine, St. George, GRD

**Keywords:** iud anxiety, women under 49, iud pain management, acute pain iud, iud insertion, intrauterine device

## Abstract

The discomfort and anxiety associated with the intrauterine device (IUD) insertion process is a significant barrier to its adoption as a form of contraception despite its high efficacy. This study aimed to classify and identify methods for minimizing pain and anxiety in IUD implantation in women below the age of 49. A search of publications from online databases, PubMed and Google Scholar, revealed 14 articles that met the inclusion criteria. An analysis of the selected studies showed that several pharmacological and non-pharmacological measures effectively minimized patient discomfort associated with IUD insertion. Of the 14 studies, 12 evaluated pharmacological methods for pain management in IUD insertion, while two studies assessed non-pharmacological methods. The results showed that although the IUD is more effective than other forms of contraceptives, fear of pain related to the insertion process is one of the most significant barriers to the use of an IUD among women. Most studies identified pharmacological methods of pain management for IUD insertion, highlighting a need for more research on non-pharmacological methods to improve patient experiences and reduce associated fears.

## Introduction and background

Despite its high efficacy, the discomfort and anxiety surrounding intrauterine device (IUD) insertion pose significant barriers to its widespread adoption as a contraceptive method among women under the age of 49. This study identifies various pharmacological and non-pharmacological interventions aimed at minimizing pain and anxiety during the IUD insertion process. It underscores the imperative for further research on interventions to enhance the patient experience and foster greater acceptance of the IUD.

Contraceptive methods have evolved significantly, with various methods competing for prominence in family planning. Among these, the IUD stands out as a globally used contraceptive method, with more than 14% of women opting for this form of contraception [1]. Despite its widespread use, one aspect that demands examination and improvement is the acute pain associated with IUD insertion, particularly among young women under the age of 49.

While the IUD is a highly effective contraceptive method, fears related to pain during insertion have been identified as a nearly universal barrier, hindering its acceptance, especially among younger women who experience higher rates of unintended pregnancies, which is linked to a variety of adverse outcomes such as financial strains, mental health disorders, increased likelihood of abortions, and increased infant and maternal mortality rates [2,3]. This fear stems from a lack of understanding or misinformation about the procedure, leading to anxiety and reluctance [4].

Furthermore, there is significant fear related to the potential expulsion of the IUD. Concerns about the device shifting or being expelled from the uterus create further apprehension about the reliability and effectiveness of the contraceptive method. This contributes to a lack of awareness about the low expulsion rates associated with the modern IUD [5]. Furthermore, the fear of having a foreign body in the uterus also contributes to hesitancy among adolescents considering an IUD [4]. The perception of an IUD as a foreign object within the body may trigger discomfort and anxiety, impacting the decision-making process. In addition, there is a fear of potential physical harm caused by the IUD. Women may harbor concerns about the safety of the device and the possible risks associated with its presence in the uterus. Misconceptions or lack of information regarding the safety profile of long-acting reversible contraception (LARC) further prevent the adoption of the IUD as a contraceptive [4].

Concerns surrounding the risks of IUDs have also declined their popularity. Despite this, studies have shown that the most severe complications, such as ectopic pregnancy and pelvic inflammatory disease (PID), are rare, affecting less than 1% of users across all age groups and types of IUDs. Instances of uterine perforation, while possible, are infrequent. Interestingly, young women between 15 and 19 years of age exhibit a higher likelihood of experiencing dysmenorrhea or amenorrhea within a year of IUD insertion compared to those aged 25 to 44 [6].

Addressing these fears is crucial for improving the acceptability and uptake of LARC among young women. Education and counseling aimed at dispelling misconceptions, providing accurate information about the insertion process of an IUD, addressing concerns about pain and potential harm, and highlighting the safety and efficacy of the modern IUD can play a vital role in overcoming these barriers and promoting informed decision-making among young women [7].

## Review

Methods

**Figure 1:**
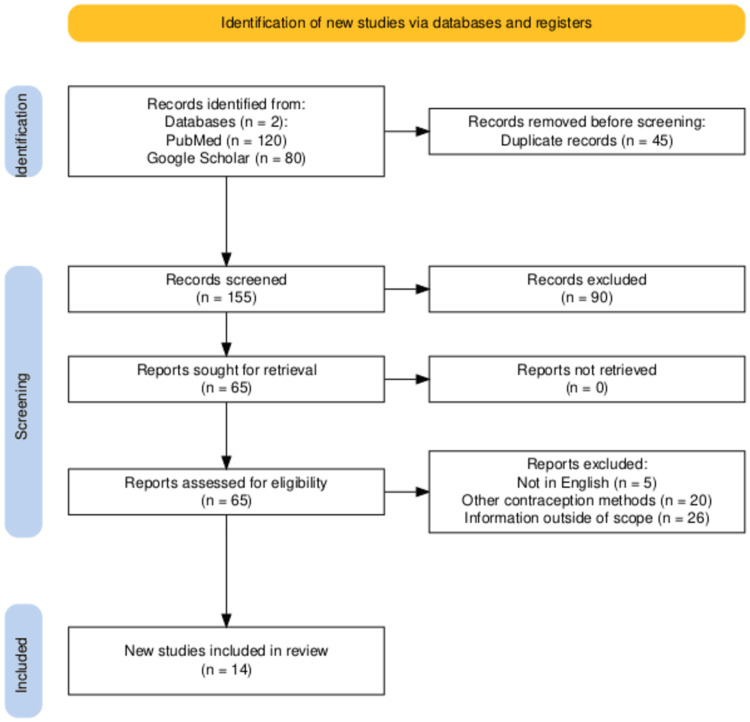
PRISMA flow diagram PRISMA, Preferred Reporting Items for Systematic Reviews and Meta-Analyses

To identify relevant studies, a systematic search was conducted using two primary databases, PubMed and Google Scholar. The search used specific keywords and Boolean operators in Table 1 to ensure comprehensive retrieval of pertinent literature. The search terms included combinations of the following keywords: “intrauterine device,” “IUD insertion,” “acute pain IUD,” “IUD pain management,” “women under 49,” and “IUD anxiety.” The initial search yielded 200 articles (120 from PubMed and 80 from Google Scholar). Forty-five duplicate articles were identified and removed. Titles, abstracts, and discussion sections of 155 articles were reviewed to assess the relevance of the articles. Peer-reviewed articles and reports published in the English language targeting women in several countries, including relevant information on inserting an IUD and pain management strategies, were selected for analysis. Eligible publications were analyzed between 2012 and 2023 with free access to the full text. The study population included women of childbearing age, described as women under 49 years of age. Articles were excluded if they were not in English, focused on other contraception methods, or contained information on the IUD outside the scope of this study. Fourteen articles met all inclusion criteria and were included in the final review and analysis. Figure 1 shows a Preferred Reporting Items for Systematic Reviews and Meta-Analyses (PRISMA) flow diagram through the different phases of the systemic review.

**Table 1:** Search terms and keywords

Search Terms	Keywords
“intrauterine device pain,” “intrauterine device insertion,” intrauterine device anxiety,” “intrauterine device pain management”	intrauterine device pain, IUD insertion, acute pain IUD, IUD pain management, women under 49

Results

The present review summarized and analyzed 14 publications listed in Table 2. The network meta-analysis showed that most articles offered pharmacological intervention before IUD insertion. Providers across multiple articles in various countries recommended ibuprofen 800mg one hour before the procedure along with a local anesthetic. These methods reduced the pain experienced by patients. However, they did not address the issue of pre-procedure anxiety as a result of fear of pain of IUD insertion, which raises bioethical concerns in the patient-physician relationship [8].

**Table 2:** Literature summary IUD, intrauterine device; LARC, long-acting reversible contraception; VAS, visual analog scale

Reference	Study Design	Treatment(s) Administered	Study Population	Findings
Daniels and Abma, 2020 [8]	Survey	N/A	Women aged 15-49 in the US	Contraception method use between 2017 and 2019 in the US: female sterilization 18.1%, oral contraceptive pills 14.0%, LARCs 10.4%, and male condom 8.4%
Hunter et al., 2020 [4]	Single-blind randomized trial	1% lidocaine paracervical block	Adolescents and young adult women aged 14-22	Anticipated pain is positively associated with perceived pain during IUD insertion. Black participants had a higher anticipated VAS score.
Cirik et al., 2013 [9]	Single-blind randomized trial	1% lidocaine paracervical block	Turkish women aged 18-45	Lower perception of pain in the 1% lidocaine group compared to saline and no treatment
Lopez et al., 2015 [10]	Literature review	N/A	N/A	The most compelling evidence of interventions for pain control comes from single trials of moderate quality
Aksoy et al., 2015 [11]	Double-blind, randomized clinical trial	10% lidocaine spray	Parous women aged 19-49	No statistically significant difference was found between both groups
Akers et al., 2017 [12]	Single-blind randomized controlled trial	10-mL 1% lidocaine paracervical nerve block	Adolescents and young adult women aged 14-22	10-mL 1% lidocaine paracervical nerve block reduced pain during IUD insertion
Mody et al., 2012 [13]	Randomized controlled clinical trial	1% lidocaine paracervical block	Women seeking an IUD	1% lidocaine paracervical block was not clinically significant in decreasing perceived pain
Tavakolian et al., 2015 [14]	Triple-blind clinical trial	Lidocaine-prilocaine cream	Women in a clinic in Hamedan	Lidocaine-prilocaine cream significantly reduced pain in all steps of IUD insertion
Abbas et al., 2017 [15]	Randomized double-blind controlled trial	Lidocaine-prilocaine cream	Parous women	Lidocaine-prilocaine cream reduced VAS pain scores during all steps of IUD insertion
Lathrop et al., 2013 [16]	Double-blind randomized controlled trial	Buccal misoprostol	Nulliparous women	Buccal misoprostol did not reduce pain scores or improve ease of insertion
Scavuzzi et al., 2013 [17]	Double-blind, randomized clinical trial	Vaginal misoprostol	Nulligravid women of reproductive age	Misoprostol was clinically significant in reducing VAS pain scores
Karabayirli et al., 2012 [18]	Double-blind, randomized clinical trial	Oral tramadol and naproxen sodium	Multiparous women aged 18-49	Both tramadol and naproxen significantly reduced pain scores. However, tramadol was more effective than naproxen.
Shahnazi et al., 2012 [19]	Randomized controlled clinical trial	Lavender	Married women aged 15-49 from Bahonar Health Care Center, Iran	Inhaling lavender reduced anxiety related to IUD insertion
Nguyen et al., 2020 [20]	Literature review	N/A	N/A	The effectiveness of pharmacological techniques for pain reduction during IUD insertion varies, and non-pharmacological techniques were not clinically significant

In a single-anonymized randomized trial among women aged 14 to 22 in three family clinics in Philadelphia, Pennsylvania, researchers measured anticipated pain using a 100mm visual analog scale (VAS). Univariate analysis identified race and age as contributors to the expected pain of IUD insertion. White participants had a median anticipated pain of 51 (interquartile range [IQR] 35-68), Black or African American participants had a median expected score of 68 (IQR 52-83), and other races had a median anticipated pain score of 64 (IQR 36-78) (p=0.012) [4]. Younger participants aged 14 to 17 had a median anticipated score of 69 (IQR 46-86), while older participants aged 18 to 22 had a median anticipated pain score of 59 (IQR 36-71) (p=0.016) [4]. Further studies show that Black participants had an anticipated VAS pain score that was 15.1mm higher on average compared to White participants (p=0.017). Values based on race and age were statistically significant but may not be clinically significant [4].

Pharmacological Interventions

Twelve studies examined lidocaine, misoprostol, nonsteroidal anti-inflammatory drugs (NSAIDs), and other pharmacological interventions to relieve pain and anxiety during IUD insertion [9-20]. Lidocaine 1% (10mL) paracervical block significantly decreased the pain when compared to placebo during IUD insertion and reduced pain after IUD insertion (5min) compared to placebo [9]. Lidocaine 2% gel reduced VAS pain scores by a clinically significant amount during IUD insertion compared to the placebo group [20], and further research is unlikely to change the confidence interval in the estimate of effect. Lidocaine 4% topical gel (8.5mL) significantly decreased pain compared to placebo immediately after application for IUD insertion [10]. Lidocaine 10% spray (40mg) topically applied dramatically reduced pain during IUD insertion immediately after IUD insertion when compared to placebo. The mean pain scores before speculum placement and immediately following IUD insertion were 1.01 ± 1.20 for the lidocaine spray group and 3.23 ± 1.60 for the placebo spray group, with a statistical significance of p<0.001 [11,20].

The effectiveness of 10mL of 1% lidocaine gel in relieving pain was inconclusive, with one study finding clinically significant results in median VAS score after IUD insertion in the lidocaine block group versus the control group (30.0 [95% CI 20.0-58.0] vs. 71.5 [95% CI 66.0-82.0]; p<0.001), and the other study found no significant difference between the groups [12, 20]. When comparing the sham block group to the lidocaine block group, the median difference between VAS scores recorded at block placement and IUD insertion was substantially larger (p=0.001) [12]. Nevertheless, in the lidocaine block group, there is no difference in the median VAS scores between these two procedural steps (p=0.31) [12]. Analysis of the secondary outcomes found that the VAS scores across all six VAS assessments were lower in the lidocaine block group compared with the sham block group in the unadjusted (27.7 [95% CI 16.0-40.2] compared with 53.9 [95% CI 44.0-57.8], p=0.001) and adjusted ranked analysis of covariance of p=0.001 [12,13].

Lidocaine and prilocaine (EMLA cream) 5% (5g) significantly decreased pain compared to placebo during IUD insertion immediately after the procedure (p<0.001) [14,15,20]. In a double-blind, randomized controlled trial, half of the patients received 2mL EMLA cream applied to the anterior cervical lip and 2mL EMLA cream applied into the cervical canal. The experimental group reported clinically significant lower pain scores (p=0.0001) during the insertion of the IUD, placement of the tenaculum, and insertion of the uterine sound [15].

Misoprostol (400ug), when applied vaginally, reduced the VAS pain score by a clinically significant amount during the IUD insertion procedure compared to placebo [16,17,20]. However, in one trial, Misoprostol (400ug) applied buccally significantly increased pain, such as cramping, compared to placebo during IUD insertion, and increased pain after IUD was also noted before clinic departure [16], warranting more research on the efficacy of misoprostol in reducing pain.

Tramadol 50mg, tablet oral, significantly decreased pain compared to naproxen 550mg during IUD insertion (2.31 ± 0.60 [95% CI 2.09-2.53] vs. 4.88 ± 1.0 [95% CI 4.54-5.22]; p = 0.001). Naproxen 550mg, tablet oral, significantly decreased pain during IUD insertion compared to placebo. However, the tramadol group significantly reduced pain scores compared to the naproxen group (p=0.003), and the naproxen group had significantly reduced pain scores compared to the control group [18].

The use of topical agents (40mg 10% lidocaine spray, 10mL 1% lidocaine gel, and 2mL 5% EMLA cream) and 50mg oral tramadol clinically decreased VAS pain scores significantly during the IUD insertion process compared to the placebo group. Patients’ overall pain throughout the treatment was significantly reduced by using 40mg of 10% lidocaine spray (average pain scores before speculum placement and immediately after IUD insertion) [20].

Studies revealed that the most clinically significant predictor of pain at IUD insertion (p < 0.001) was negative perceptions of IUDs, even though cesarean birth and pre-procedure anxiety were linked to greater pain scores [7]. Previous vaginal delivery under epidural analgesics showed a correlation with lower pain scores at IUD insertion (p<0.001). Moreover, women with mild anxiety had significantly higher mean VAS pain scores in women with mild anxiety than women with minimal anxiety before examination and throughout the procedure [7]. Negative perceptions of IUD were high among women with dysmenorrhea and women with mild anxiety (both p<0.001). Women who experienced less fear were substantially more likely to have higher parity and have had an IUD insertion experience (p <0.001 and p <0.05, respectively) [7]. Anticipated pain was also positively associated with pain at IUD insertion (p<0.001) [12].

A univariate analysis identified race and age as contributors to the anticipated pain of IUD insertion. White participants had a median expected pain of 51 (IQR 35-68), Black or African American participants had a median anticipated score of 68 (IQR, 52-83), and other races had a median anticipated pain score of 64 (IQR 36-78) (p=0.012) [4]. Younger participants between 14 and 17 years of age had a median anticipated score of 69 (IQR 46-86), while older participants between 18 and 22 years of age had a median anticipated pain score of 59 (IQR 36-71) (p=0.016) [4]. Further studies show that Black participants had an anticipated VAS pain score that was 15.1mm higher on average compared to White participants (p=0.017) [4].

Non-Pharmacological Interventions

Very few studies retrieved measured anxiety before IUD insertion. In a randomized controlled clinical trial, researchers found that inhaled lavender, compared to placebo on pain and anxiety during IUD, had significantly decreased mean anxiety scores after the use (compared with before the use of lavender) (43.2 ± 9.2 to 39.0 ± 10.5; p< 0.001); however, the levels of pain after IUD insertion in the intervention and placebo groups were not significantly different [19, 20]. Other non-pharmacological interventions include applying vulsellum slowly versus quickly, applying it during, before, or after the menstrual cycle, inhaling 50% nitrous oxide and 50% oxygen versus oxygen, using a dental armamentarium to apply paracervical block analgesia, and applying paracervical block anesthesia with a jet injector rather than needle and syringe [20]. However, none of these non-pharmacological techniques had clinically significant results [20].

Discussion

Pain management strategies encompass pharmacological and non-pharmacological interventions, each offering advantages and limitations. Among the studies retrieved, the most significant pharmacological methods of mitigating pain during IUD insertion were lidocaine, misoprostol, NSAIDs, and tramadol. These aim to target pain directly and provide immediate relief during and after IUD insertion. These medications have shown efficacy in reducing pain scores, with lidocaine demonstrating significant decreases in pain compared to placebo. Some pharmacological interventions, such as misoprostol, may lead to increased discomfort due to associated side effects, such as cramping. When comparing tramadol and NSAIDs like naproxen, tramadol outperforms naproxen in reducing pain, but naproxen is more effective than placebo. Furthermore, combining tramadol with a topical agent such as lidocaine or EMLA cream reduces pain throughout the procedure. These findings suggest that a multimodal pain management approach combining systemic and topical interventions can enhance patient comfort during IUD insertion.

While pharmacological interventions alleviate the physical pain experienced during the procedure, the explored non-pharmacological interventions aim to induce relaxation and relieve any accompanying stress. According to the results, patients who harbor anxiety or negative feelings regarding IUD insertion tend to experience more significant pain. Interventions such as inhaled lavender help patients feel more at ease before the procedure and may help eliminate preconceived perceptions of pain. Further research may be warranted to investigate the efficacy of other popular herbal agents, such as lavender, inducing relaxation during IUD insertion [19]. Ultimately, a comprehensive approach that combines pharmacological and non-pharmacological interventions tailored to the patient's needs and preferences may offer the most effective pain relief.

According to the Centers for Disease Control and Prevention (CDC), approximately 65% of women used some form of contraception between 2017 and 2019. The most commonly used contraceptive method is sterilization, followed by oral contraceptive pills, LARC, and male condoms [8]. Concerns about pain during the insertion procedure primarily influence lower rates of IUD use among young women. Researchers found that negative perceptions and anxiety surrounding IUD insertion hindered the use of an IUD; increasing education on IUD insertion processes among young women may enhance the adoption of IUDs and better prevent unplanned pregnancies. The results of the present study can promote the use of an IUD, which is known to lower the rates of unplanned pregnancies, births, and abortions among young women and reduce patient anxieties related to pain by demonstrating the efficacy of pain management options for young women [12,20].

Addressing pain and anxiety during IUD insertion poses multifaceted challenges that demand comprehensive strategies. Pharmacological interventions such as lidocaine, misoprostol, and NSAIDs effectively alleviate pain; however, they often overlook pre-procedural anxiety, which plays a significant role in predicting a patient's perception of pain. Furthermore, variations in pain perception based on demographic factors such as race and age may complicate efforts to standardize pain management among patient groups. Additionally, inconclusive evidence regarding interventions such as lidocaine gel or tramadol necessitates further research. While non-pharmacological interventions such as inhaled lavender show promising results, additional research may be warranted to elucidate its efficacy among diverse populations. Moreover, the impact of previous experiences and perceptions on pain scores highlights the need for a more individualized approach to pain management. Further research in the form of randomized controlled trials and cohort studies integrating pharmacological and non-pharmacological interventions is needed to navigate these barriers.

Clinician and patient education are vital to improving LARC's current strategies. While obstetricians and gynecologists should offer appropriate contraceptive options to all patients, some clinicians are overly restrictive when considering IUD methods, frequently excluding nulliparous or adolescent patients [21]. Clinicians were also unfamiliar with the practice of immediate post-abortion and postpartum IUD insertion [22]. Continuing medical education-accredited LARC training for clinicians can sustain improvements in attitudes, clinician knowledge, and patient counseling [23]. More barriers for nulliparous and adolescent patients considering contraception are their unawareness or discomfort with IUDs, lack of parental approval, cost of insertion and the device, and unfamiliarity with the healthcare provider establishing care. Research has shown that educating the patient on numerous methods of contraception available with no worry of cost can counteract these barriers. With appropriate patient education, 67% of women chose a form of LARC, with 56% of these women choosing an IUD as their form of contraception [22].

## Conclusions

Women undergoing the IUD insertion procedure may anticipate heightened levels of discomfort, attributed to factors such as negative perceptions surrounding LARCs, pre-procedural anxiety, and apprehension regarding the pain associated with IUD implantation. Notably, a correlation exists between decreased pain experienced during IUD insertion and positive prior encounters with vaginal interventions, particularly vaginal delivery under epidural anesthesia. This association serves as a robust predictor of diminished reported pain among women. Effective management of pain during IUD insertion necessitates multifaceted approaches, including patient education aimed at rectifying misconceptions surrounding IUDs, as well as comprehensive counseling sessions aimed at elucidating the advantages and drawbacks of IUD employment, thereby mitigating pre-procedural anxiety.
